# Bacterial infections in patients with nipple piercings: a qualitative systematic review of case reports and case series

**DOI:** 10.3205/id000080

**Published:** 2022-03-30

**Authors:** Luis M. Acuña-Chávez, Christian A. Alva-Alayo, Giamfranco A. Aguilar-Villanueva, Kevin A. Zavala-Alvarado, Cristhian A. Alverca-Meza, María M. Aguirre-Sánchez, Anyelo A. Amaya-Castro

**Affiliations:** 1 Facultad de Medicina, Universidad Nacional de Trujillo, Peru; 2 Sociedad Cientifica de Estudiantes de Medicina de la Universidad Nacional de Trujillo, Peru

**Keywords:** bacterial infections, breast abscess, nipple piercing, MeSH-NLM

## Abstract

The main objective of this review is to identify the most frequently isolated bacteria in patients with infections related to nipple piercings in case reports and case series. In addition, the aim is to describe clinical manifestations and antecedents. There is a protocol of this review. The terms “bacterial infections”, “nipple piercing” and their synonyms were considered. Pubmed/Medline, Scopus, Embase, Web of Science core collection and Ovid/Medline databases were searched until November 15, 2021 without date or language restrictions. Two authors extracted the articles and three other authors performed the selection, first by title and abstract, and second by full-text revision. Discrepancies were resolved with yet two other authors. Quality was assessed using the Joanna Briggs checklists. Finally, data extraction was realized. A total of 1,531 articles were extracted, of which 20 articles were included, and one article was added by hand-searching. The final number of articles included was 21, all of them with acceptable quality of evidence. Twenty-seven patients were considered (23 women and 4 men), aged between 15–60 years old. The most frequent bacterial genus in case reports and case series was *Staphylococcus* (n=10), and the most frequent species was *M. fortuitum* (n=6), although etiology seems to be diverse. The breast was the main affected organ, and the most frequent findings were fluid collection, pain, erythema, granulation tissue and swelling. The suspicion of infection by this bacterial species could be taken into account when it is associated with nipple piercings; however, larger studies are required to give a conclusion based on the evidence.

## Introduction

Piercing is a type of body modification performed by inserting a large gauge needle through skin or cartilage, creating a fistula-like opening, usually adorned [1]. A survey in the United States reported that 35% of participants claimed to have had piercings, and 14% in places other than the earlobe [2]. Additionally, a survey conducted in France showed that people aged 25 to 34 had the highest prevalence of having a piercing, with greater frequency in women [3]. The most common visible locations for those perforations are the face, nose and ears; the semi-visible areas are the navel and tongue; and not-visible, such as nipples and perineum, have become common types of body art in both genders [4].

Nipple piercings (NP) can cause both non-infectious and infectious complications. Non-infectious complications include injuries when playing contact sports, galactorrhea when nipples are stimulated, etc. [5]. On the other hand, NP could favor the access of pathogens that lead to local infections at the area of perforation that could spread to surrounding tissues, causing mastitis or abscesses [6].

The isolation of the specific type of bacteria could be essential to choose the most appropriate treatment. In general, the therapeutic approach for breast abscess recommends accompanying the drainage with antibiotics focused on the suspicion of *S. aureus* [7]. However, the presence of NP may predispose to infections caused by other types of pathogens. There are systematic reviews about etiology and complications from ear cartilage [8], tongue [9] and lip [9] piercings. However, to date there is no systematic review about the complications or etiology of NP, the incidence of which appeared to be 21% [10]. Additionally, the presence of NP is significantly associated to the development of breast abscess [11], a clinical manifestation of bacterial breast infections. Therefore, in order to answer a specific question with evidence-based methodology, this systematic review focuses on bacterial infections in patients with nipple piercings. Nowadays, the main source of information about bacterial infections associated with NP are case reports and case series. Consequently, in order to determine which are the most commonly isolated bacteria in these patients, these types of publications have been critically reviewed in this article. In addition, clinical manifestations and antecedents are described.

## Materials and methods

Case reports and case series about bacterial infections in the mammary region, heart, skin or blood in patients with an antecedent of NP were searched. In this review, “case series” are defined as those studies in which more than 5 cases are reported [12]. There is a pre-published protocol of this systematic review registered in PROSPERO, CRD42021236900 (https://www.crd.york.ac.uk/prospero/display_record.php?ID=CRD42021236900). This review was conducted in accordance with PRISMA [13] (Attachment 1, Supplementary Material 1).

### Data sources and search

PubMed, Scopus, Embase, Web of Science core collection and Ovid/Medline databases were searched until November 15, 2021. The terms “bacterial infections”, “nipple piercing” and their synonyms were considered; however, terms referring to piercings in other body locations were excluded. There were no date or language restrictions. The PubMed search strategy was modified for use in other databases (Attachment 1, Supplementary Material 2). Additionally, a hand-search was performed in the same databases to identify other potentially relevant articles.

### Selection criteria

The inclusion criteria were: 1) having an NP; 2) bacterial infection in the mammary region, associated skin, heart or blood; and 3) isolation and identification of the bacterial genus and species. The following exclusion criteria were considered: 1) incorrect population: patients infected by other types of microorganisms (viruses, fungi, parasites) or patients without NP; 2) incorrect publication type: revisions, misprints, etc.; 3) not having access to full-text; and 4) reports that did not specify causative agent. In addition, not all patients from every selected article were included; in contrast, just those patients in which the infection-causing bacteria was identified were included, defined as “eligible cases”.

### Selection of studies

Two authors (GAAV, MMAS) exported the articles from the databases to Rayyan software (https://www.rayyan.ai/). Then, duplicates were removed to continue with the selection, carried out independently by three authors (LMAC, KAZA, CAAM), first by title and abstract, and second by full-text revision. Discrepancies were resolved with two other authors (CAAA and AAAC).

### Data extraction

Data were extracted and verified by all the authors. The following data were extracted: 1) author; 2) age and sex of the patient; 3) compromised nipple; 4) the length of time the patient had the NP; 5) clinical presentation and antecedents; and 6) isolated bacteria and treatment in each case.

### Quality assessment

The quality was assessed using the Joanna Briggs Institute checklist for case reports [14] and for case series [15]. Acceptable quality was considered for cases that satisfied 5 appraisal items [16].

## Results

### Selected studies

**Figure 1:**
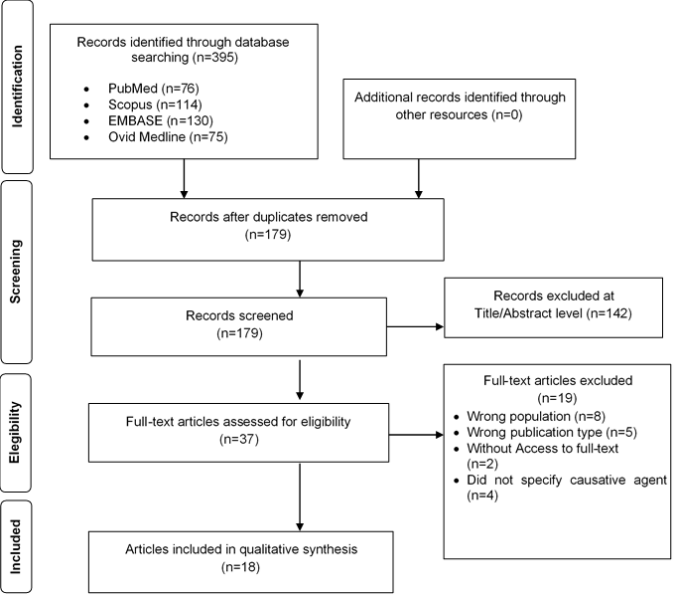
PRISMA flowchart

A total of 1,531 articles were extracted from PubMed (n=175), Scopus (n=444), Embase (n=486), Web of Science core collection (n=137) and Ovid/Medline (n=288). Additionally, one article was added by hand-searching in the five electronic databases mentioned [17]. Removal of duplicate articles resulted in a total of 488. In the selection by title and abstract, 431 articles were eliminated. With the remaining 57, a full-text review was carried out, in which 36 articles were excluded for the reasons given in Figure 1 (Fig. 1), where the flowchart of the selection process according to PRISMA is shown [13]. Finally, 21 articles were considered in this review [17], [18], [19], [20], [21], [22], [23], [24], [25], [26], [27], [28], [29], [30], [31], [32], [33], [34], [35], [36], [37].

### Characteristics of the selected studies

Twenty-one articles were included for qualitative synthesis, all of them with acceptable quality of evidence (Attachment 1, Supplementary Material 3). Four out of 21 articles were case series, and the rest were case reports. Regarding the case reports, one of them presented three eligible cases [19]; on the other hand, three of the case series presented more than one eligible case: one of them presented three [27] and the other, four [30]. All other articles only presented one eligible case, such that 27 patients were considered in total. The following data is summarized for each eligible case: 1) patient characteristics; 2) clinical presentation and antecedents; and 3) isolated bacteria. Additional information can be found in Table 1 (Tab. 1).

### Patient characteristics

Of the 27 patients, 23 were women [17], [19], [20], [23], [24], [25], [27], [28], [29], [30], [31], [32], [33], [34], [35], [36], [37] and 4 were men [18], [21], [22], [26], with an age range between 15–60 years old. With regard to the piercing location, 15 patients had the piercing in the right nipple [17], [18], [20], [21], [23], [25], [27], [28], [29], [31], [34], [35], [36], 7 in the left nipple [19], [22], [24], [26], [32], [33], and 1 in both nipples [37]; however, in four patients this information was not described [30]. The time between the placement of the piercing and the infection was not specified in nine cases [18], [24], [28], [30], [33]; on the other hand, regarding those that were specific: 3 patients had NP for a period less than 1 month [17], [23], [27]; 7 patients had NP for a period greater than 1 month but less than 6 months [19], [22], [25], [26], [29], [34], [37]; 6 patients had NP for a period greater than or equal to 6 months but less than or equal to 1 year [19], [20], [21], [27], [32], [36]; and only 3 patients had NP for more than 1 year [27], [31], [35].

### Clinical presentation and antecedents

The breast was the main affected organ in the clinical presentation. Breast fluid collection was found in 22 patients [17], [18], [19], [20], [21], [23], [24], [25], [26], [27], [28], [29], [31], [32], [33], [34], [35], [36], breast pain or tenderness in 10 patients [18], [19], [23], [25], [27], [28], [32], [34], [35], [36], breast enlarging or swelling in 9 patients [17], [18], [19], [21], [25], [26], [27], [34], breast erythema in 8 patients [17], [18], [19], [25], [26], [31], [32], [34], and granulomatous tissue in 5 patients [19], [24], [28], [36], [37]. The following findings were not reported in more than one patient: chest wall cellulitis [22], retroareolar cellulitis [21], dyspnea and productive cough with bloody sputum [22], hyperpigmentation [24], breast induration [17] and endocarditis [22].

Some antecedents were reported, as follows: sexual contact with possible exposure of the pierced nipple [21], [31], smoking [19], [29], [35], breast implants [19], [32], pectoral and calf implants [18], exposure or swimming in dirty water [33], or in the ocean [24], [26], touching the nipple with objects [35] and the presence of prosthetic aortic valve [22]. Clinical presentation and antecedents of the patients are summarized individually in Table 2 (Tab. 2).

### Isolated bacteria

The most frequently isolated bacterial genera were *Staphylococcus* (n=10) and *Mycobacterium* (n=9), all in different patients except for two of them [19], [34]. All isolated *mycobacteria* were non-tuberculous *mycobacteria* (NTM). In total, there were 6 cases of infection due to *M. fortuitum* [24], [25], [26], [29], [33], [37]; 8 cases due to coagulase-negative *Staphylococcus* [19], [22], [32], [34], [35], 4 of them confirmed as *S. epidermidis* [22], [32], [34], [35]; 2 due to *N. gonorrhoeae* [21], [31]; 2 due to *S. aureus* [23], [27]; 2 due to *S. agalactiae* [19], [27]; and 2 due to *P. acnes* [28], [30]. In addition, the following bacteria were identified in one patient only: *A. turicensis* [35], *G. terrae* [20], *P. melanogenica* [25], *P. intermedia* [17], *P. anaerobius* [17], *Nocardia* sp. [33], *M. chelonei* [34], *P. harei* [35], *M. abscessus* [36], *M. holsaticum* [19], *M. agri* [19], *M. brumae* [19], *Actinomyces* [27], *P. acnes* [30], *C. amycolatum* [30], *H. parainfluenzae* [30], group A beta-hemolytic *Streptococcus* [18], a “green microaerophilic *Streptococcus”* [19], and a rare gram-positive coccus not otherwise specified [30]. On the other hand, some cases corresponded to co-infections, for example: *M. fortuitum* was reported as coinfection in two cases, in one of them with *P. melanogenica* [25] and in the other case with *Nocardia* sp. [33]; *S. epidermidis* was also reported as coinfection in two cases, in one of them with *M. chelonei* [34] and in the other case with *Actinomyces* and *P. harei* [33]; also, one of the *S. agalactiae* cases was actually a co-infection with coagulase-negative *Staphylococcus* [19]; moreover, *P. intermedia* was reported with *P. anaerobius* [17]; finally, there was a co-infection of *C. amycolatum*, *P. acnes* and *H. parainfluenzae* [30]. Isolated bacteria, as well as treatments for each patient are summarized in Table 3 (Tab. 3).

## Discussion

Cases of breast abscess in non-lactating patients usually present a combined flora of *S. aureus*, *Streptococcus*, and anaerobic bacteria [38]. In this review, the most frequent bacterial genus was *Staphylococcus* (n=10); followed by *Mycobacterium* (n=9), specifically coagulase-negative *Staphylococcus* and NTMs, respectively; and the most commonly identified species were *M. fortuitum* (n=6) and *S. epidermidis* (n=4).

The fast-growing mycobacteria, or Runyon’s group IV, are the most common cause of soft tissue Mycobacterium infection and they are often related to trauma [39]. According to the literature, infections by *M. fortuitum* (a fast-growing type of mycobacterium) appear to be unusual [40]. *M. fortuitum* usually causes skin and soft tissue infections after direct inoculation, such as in trauma, surgery or cosmetic procedures; although, according to literature, it seems to be less frequent in the latter [41]. However, *M. fortuitum* was the most frequently isolated bacterial species in this review. In addition, *M. fortuitum* has also been isolated in infections related to other cosmetic procedures besides piercings, such as pedicures [42], [43], tattooing [44], and mesotherapy [45]. Five of the six cases of infection by *M. fortuitum* reported fluid collection. This fact is corroborated in the literature, since in most cases this type of bacteria causes pustules, nodules with or without suppuration, granulomas with the presence of a central or necrotic caseous area and a sporotrichoid pattern, with susceptibility to certain antibiotics, such as amikacin, clarithromycin, azithromycin, erythromycin, cefoxitin, and doxycycline [46].

*Staphylococcus* was the most frequent bacterial genus found in this review. *S. aureus* can cause localized inflammation, cellulitis, or even the formation of abscesses, which begin as a localized acute inflammatory response [47]. In fact, according to literature, most primary breast abscesses are associated with *S. aureus* infection, therefore empirical antibiotics are usually based on suspicion of this bacterium [7]. However, in this review, *S. aureus* was reported less frequently than *S. epidermidis*, which was isolated in 4 patients (although coagulase-negative *staphylococcus* was reported in 8 patients). Nevertheless, it should be noted that *S. epidermidis* was not the main cause of breast infection in two of these patients [34], [35]. *S. epidermidis* is a harmless commensal of the skin and mucous membranes; however, this pathogen can also cause infection from exogenous sources, such as endocarditis from native and prosthetic valves [48], catheter surfaces [49], and medical implants [50]. The latter has been reported in this review. One of the included articles reports a case of *S. epidermidis* infection in a patient with history of bilateral augmentation with silicone implants two years before placing the NP in both nipples [32]. In addition, there was a similar case with pectoral and calf implants; however, in this patient group, A beta-hemolytic *streptococcus* was isolated [18]. Additionally, another of the reviewed cases reports a native valve endocarditis due to the spread of *S. epidermidis* from a previous mastitis [22]. Since *S. epidermidis* is a human commensal, all of the case reports suggest a contamination by the normal flora of the skin in which the access point is the hole created by the NP.

Two cases of *N. gonorrhoeae* infection were also identified. These two patients reported recent sexual contact involving the NP, one with the partner’s penis [31], and the other with the mouth [21]. *N. gonorrhoeae* can be easily transmitted from men to their sexual partners, since it can adhere to sperm, causing high bacterial concentrations in this fluid [51]. In one of these patients [31], penis-nipple contact was doubtful, but mouth-nipple contact was reported; in the same way in the other one [21], in which all types of contact with ejaculatory fluid were denied, but vigorous contact of mouth-mouth and mouth-nipple type was confirmed. Although the main transmission mechanism is through direct penile-vaginal contact or vice versa, representing approximately 70% of the cases according to literature [52], saliva could represent the transmission path in the cases presented in this review, since the presence of *N. gonorrhoeae* in saliva and pharyngeal secretions has been previously demonstrated [53].

A large number of patients were women. This seems to be related to the fact that the use of NP is more common in women, but actually literature suggests that NP is more common in men [2], [54]. However, NP could cause more problems in women than in men, since women have more adjacent subcutaneous tissue in the breast region, which represents an entry route for pathogenic organisms [40]. Obesity [19] and smoking [29], [35] were the main antecedents reported in this review. In fact, these two variables were previously identified as risk factors for the development of breast abscess [11], [55]. Moreover, it is advisable to ask the patient about the history of NP, even when this is not evident in the clinical presentation, since the patient could have removed it before [36].

It is suggested to start antibiotic treatment as soon as a bacterial skin infection is suspected, after taking a culture, then maintaining or changing the antibiotic according to the results of the antibiogram, depending on the individual case. Additionally, if the case corresponds to a breast abscess, it will be necessary to drain or aspirate the fluid. On the other hand, culture results sometimes could be negative, but because of clinical features and the casuistry found in this review, the clinician will have to assess whether to continue or suspend the antibiotic treatment. Most of the patients – 22 out of 27 total to be precise – presented fluid collection in the mammary region. The therapeutic approach guides for breast abscesses recommend accompanying the drainage with targeted antibiotics for the suspicion of *S. aureus* [7]. Although etiology in patients with NP seems to be diverse, the suspicion of infection by *M. fortuitum* could be taken into account. However, we suggest larger studies (i.e., case control studies) to confirm, based on evidence, a possible association between NP and *M. fortuitum* infection. Despite having considered thorough exclusion criteria and a critical appraisal checklist, the results of the case reports and case series by their nature are not representative for the entire population. However, as it constitutes the only evidence available about the etiology of bacterial infections in patients with NP, this systematic review provides an important first step in determining the etiology of infections among different bacterial species in patients with nipple piercings, especially if the infection persists despite the initial treatment.

## Conclusions

- The bacterial species with the highest frequency in the case reports and case series of patients with infections and NP was *M. fortuitum*.
- Despite the limitations of this review, the suspicion of infection by *M. fortuitum* could be taken into account, especially if the infection persists despite the initial treatment.
- Larger studies are needed to determine an association between NP and *M. fortuitum* infection.

## Notes

### Acknowledgments

We would like to thank Dr. María Soledad Ayala Ravelo for her supervision and guidance throughout the preparation of this manuscript.

We would also like to thank Dr. William Aguilar Urbina for guiding us with his methodological and clinical expertise in the process of drafting the discussion of results.

### Authors’ ORCIDs

- Luis M. Acuña-Chávez: 0000-0003-3953-6446
- Christian A. Alva-Alayo: 0000-0002-7056-7219
- Giamfranco A. Aguilar-Villanueva: 0000-0001-5880-056X
- Kevin A. Zavala-Alvarado: 0000-0002-8084-4582
- Cristhian A. Alverca-Meza: 0000-0001-5473-4198
- María M. Aguirre-Sánchez: 0000-0002-5584-3350
- Anyelo A. Amaya-Castro: 0000-0003-1010-2599

### Competing interests

The authors declare that they have no competing interests.

## Supplementary Material

Supplementary Material

## Figures and Tables

**Table 1:**
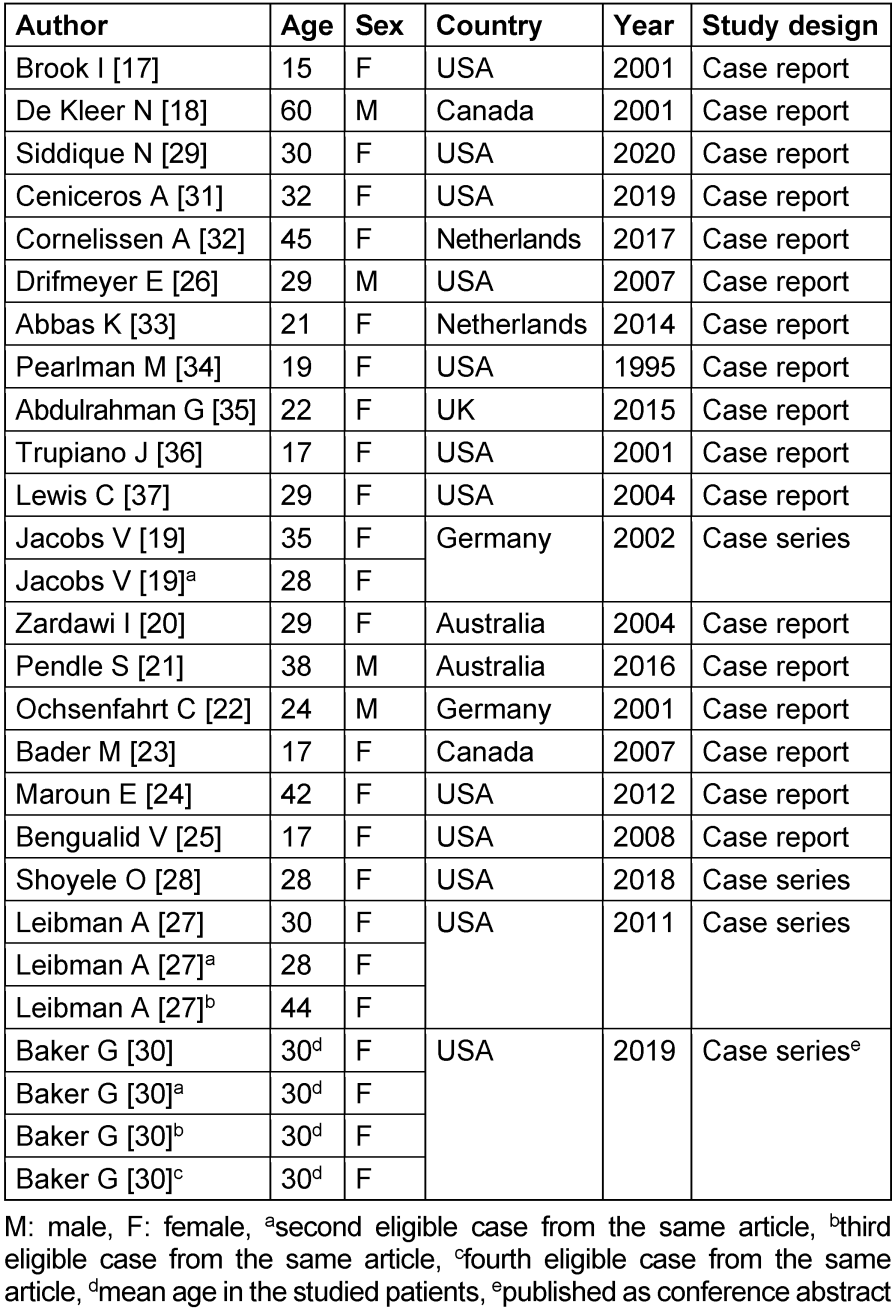
Characteristics of the included studies

**Table 2:**
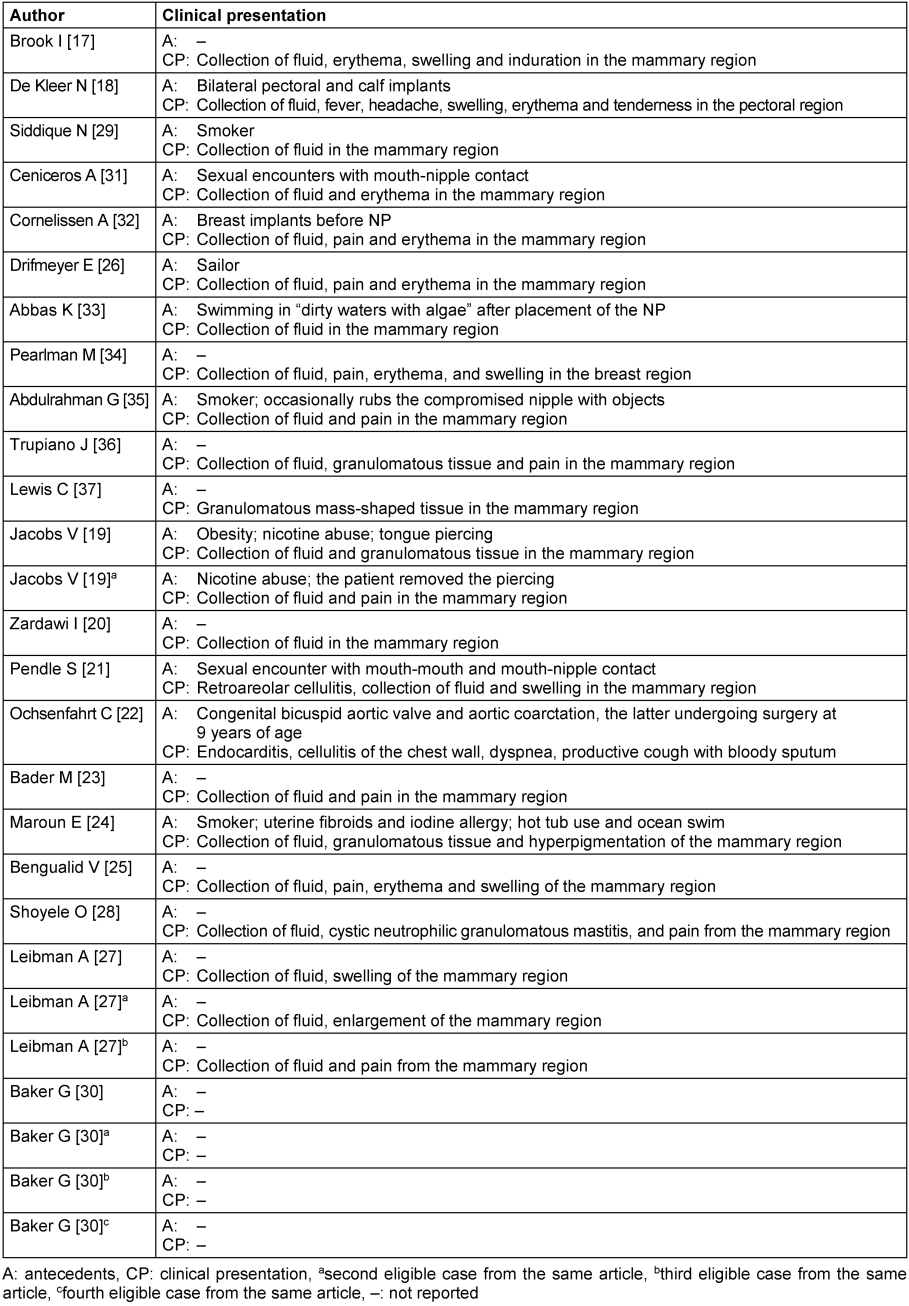
Antecedents and clinical presentation

**Table 3:**
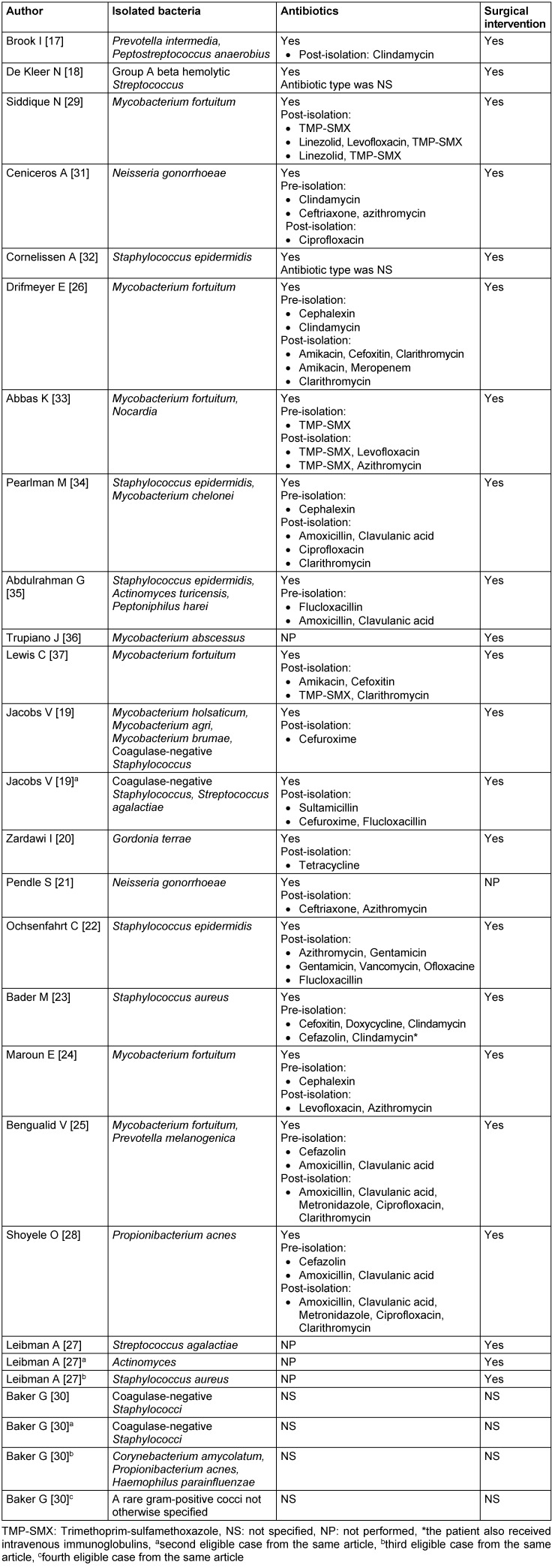
Isolated bacteria and treatment
