# Supplementary material for: Bacterial infections in patients with nipple piercings: a qualitative systematic review of case reports and case series
Source: GMS Infect Dis. 2022 Mar 30;10:Doc03. doi: 10.3205/id000080 (PMC9006427; doi:10.3205/id000080)
Supplement: Supplementary Material [file ID-10-03-s-001.pdf]

1 Supplementary Material 1: PRISMA Checklist

Section/topic	#	Checklist item	Reported on page #
TITLE			
Title	1	Identify the report as a systematic review, meta-analysis, or both	# 01 (Title)
ABSTRACT			
Structured summary	2	Provide a structured summary including, as applicable: background; objectives; data sources; study eligibility criteria, participants, and interventions; study appraisal and synthesis methods; results; limitations; conclusions and implications of key findings; systematic review registration number	# 02 (Abstract)
INTRODUCTION			
Rationale	3	Describe the rationale for the review in the context of what is already known	# 03 (Introduction)
Objectives	4	Provide an explicit statement of questions being addressed with reference to participants, interventions, comparisons, outcomes, and study design (PICOS)	# 03 (Introduction)
METHODS			
Protocol and registration	5	Indicate if a review protocol exists, if and where it can be accessed (e.g., Web address), and, if available, provide registration information including registration number	# 04 (Materials and methods)
Eligibility criteria	6	Specify study characteristics (e.g., PICOS, length of follow-up) and report characteristics (e.g., years considered, language, publication status) used as criteria for eligibility, giving rationale	# 04 (Selection criteria)
Information sources	7	Describe all information sources (e.g., databases with dates of coverage, contact with study authors to identify additional studies) in the search and date last searched	# 04 (Data sources and search)
Search	8	Present full electronic search strategy for at least one database, including any limits used, such that it could be repeated	Supplementary material 2
Study selection	9	State the process for selecting studies (i.e., screening, eligibility, included in systematic review, and, if applicable, included in the meta-analysis)	# 04 (Selection of studies)
Data collection process	10	Describe method of data extraction from reports (e.g., piloted forms, independently, in duplicate) and any processes for obtaining and confirming data from investigators	# 04 (Data extraction)
Data items	11	List and define all variables for which data were sought (e.g., PICOS, funding sources) and any assumptions and simplifications made	Protocol and #04 (Selection criteria and Data extraction)
Risk of bias in individual studies	12	Describe methods used for assessing risk of bias of individual studies (including specification of whether this was done at the study or outcome level), and how this information is to be used in any data synthesis	# 04 (Quality assessment)
Summary measures	13	State the principal summary measures (e.g., risk ratio, difference in means)	–
Synthesis of results	14	Describe the methods of handling data and combining results of studies, if done, including measures of consistency (e.g., I^2) for each meta-analysis	–

Risk of bias across studies	15	Specify any assessment of risk of bias that may affect the cumulative evidence (e.g., publication bias, selective reporting within studies)	–
Additional analyses	16	Describe methods of additional analyses (e.g., sensitivity or subgroup analyses, meta-regression), if done, indicating which were pre-specified	–
RESULTS			
Study selection	17	Give numbers of studies screened, assessed for eligibility, and included in the review, with reasons for exclusions at each stage, ideally with a flow diagram	# 05 (Selected studies) and # 20 (Figure 1)
Study characteristics	18	For each study, present characteristics for which data were extracted (e.g., study size, PICOS, follow-up period) and provide the citations	# 05 (Characteristics of the selected studies)
Risk of bias within studies	19	Present data on risk of bias of each study and, if available, any outcome level assessment (see item 12)	# 05 (Characteristics of the selected studies) and Supplementary material 3.
Results of individual studies	20	For all outcomes considered (benefits or harms), present, for each study: (a) simple summary data for each intervention group (b) effect estimates and confidence intervals, ideally with a forest plot	# 05-06 (Patient characteristics, Clinical presentation and antecedents, and Isolated bacterias), and # 15-19 (Table 2 and Table 3)
Synthesis of results	21	Present results of each meta-analysis done, including confidence intervals and measures of consistency	–
Risk of bias across studies	22	Present results of any assessment of risk of bias across studies (see Item 15)	–
Additional analysis	23	Give results of additional analyses, if done (e.g., sensitivity or subgroup analyses, meta-regression [see Item 16])	–
DISCUSSION			
Summary of evidence	24	Summarize the main findings including the strength of evidence for each main outcome; consider their relevance to key groups (e.g., healthcare providers, users, and policy makers)	# 07-08 (DISCUSSION)
Limitations	25	Discuss limitations at study and outcome level (e.g., risk of bias), and at review-level (e.g., incomplete retrieval of identified research, reporting bias)	# 08 (DISCUSSION)
Conclusions	26	Provide a general interpretation of the results in the context of other evidence, and implications for future research	# 09 (CONCLUSIONS)
FUNDING			
Funding	27	Describe sources of funding for the systematic review and other support (e.g., supply of data); role of funders for the systematic review	Protocol and #09

2 Supplementary Material 2: Search Strategies

2.1 PubMed/MEDLINE Search Strategy

Search strategy	Formulas
#1	"Body Piercing"[MH] OR "Body Piercings"[MH] OR "Piercings, Body"[MH] OR "Piercing, Body"[MH] OR "Nipple Piercing"[MH] OR "Nipple Piercings"[MH] OR "Piercing, Nipple"[MH] OR "Piercings, Nipple"[MH] OR "Body Piercing*"[TIAB] OR "Nipple Piercing*"[TIAB] OR "Body Piercing*"[OT] OR "Nipple Piercing*"[OT]
#2	"Ear Piercing"[MH] OR "Ear Piercings"[MH] OR "Piercing, Ear"[MH] OR "Piercings, Ear"[MH] OR "Lip Piercing"[MH] OR "Lip Piercings"[MH] OR "Piercing, Lip"[MH] OR "Piercings, Lip"[MH] OR "Navel Piercing"[MH] OR "Navel Piercings"[MH] OR "Piercing, Navel"[MH] OR "Piercings, Navel"[MH] OR "Tongue Piercing"[MH] OR "Piercing, Tongue"[MH] OR "Piercings, Tongue"[MH] OR "Tongue Piercings"[MH] OR "Eyebrow Piercing"[MH] OR "Eyebrow Piercings"[MH] OR "Piercing, Eyebrow"[MH] OR "Piercings, Eyebrow"[MH] OR "Ear Piercing*"[TIAB] OR "Lip Piercing*"[TIAB] OR "Navel Piercing*"[TIAB] OR "Tongue Piercing*"[TIAB] OR "Eyebrow Piercing*"[TIAB] OR "Body Piercing*"[OT] OR "Ear Piercing*"[OT] OR "Lip Piercing*"[OT] OR "Navel Piercing*"[OT] OR "Tongue Piercing*"[OT] OR "Eyebrow Piercing*"[OT] OR "genital piercing*"[TIAB] OR "oral piercing*"[TIAB] OR "nose piercing*"[TIAB] OR "genital piercing*"[OT] OR "oral piercing*"[OT] OR "nose piercing*"[OT]
#3	#1 NOT #2
#4	"Bacterial infections"[MH] OR "Infection"[MH] OR "Infections Bacterial"[MH] OR "Infection Bacterial"[MH] OR "Bacterial Disease"[MH] OR "Bacterial Diseases"[MH] OR "Bacterial Infection"[MH] OR "Bacteremia"[MH] OR "Bacteremias"[MH] OR "Bacterial infection*"[TIAB] OR "Infection"[TIAB] OR "Infection* Bacterial"[TIAB] OR "Bacterial Disease*"[TIAB] OR "Bacteremia*"[TIAB] OR "Bacterial infection*"[OT] OR "Infection"[OT] OR "Infection* Bacterial"[OT] OR "Bacterial Disease*"[OT] OR "Bacteremia*"[OT] OR "Bacteria*"[TIAB] OR "Bacteria*"[OT] OR "Endocarditis, Bacterial"[MH] OR "Bacterial Endocarditis"[MH] OR "Endocarditides, Bacterial"[MH] OR "Bacterial Endocarditides"[MH] OR "Gram-Negative Bacterial Infections"[MH] OR "Bacterial Infection, Gram-Negative"[MH] OR "Gram Negative Bacterial Infections"[MH] OR "Gram-Negative Bacterial Infection"[MH] OR "Infection, Gram-Negative Bacterial"[MH] OR "Bacterial Infections, Gram-Negative"[MH] OR "Infections, Gram-Negative Bacterial"[MH] OR "Gram Positive Bacterial Infections"[MH] OR "Bacterial Infections, Gram-Positive"[MH] OR "Bacterial Infections, Gram Positive"[MH] OR "Infections, Gram-Positive Bacterial"[MH] OR "Bacterial Infection, Gram-Positive"[MH] OR "Gram-Positive Bacterial Infection"[MH] OR "Infection, Gram-Positive Bacterial"[MH] OR "Infections, Gram Positive Bacterial"[MH] OR "Gram-Positive Bacterial Infections"[MH] OR "Skin Diseases, Bacterial"[MH] OR "Bacterial Skin Disease"[MH] OR "Disease, Bacterial Skin"[MH] OR "Skin Disease, Bacterial"[MH] OR "Bacterial Skin Diseases"[MH] OR "Mastitis"[MH] OR "Bacterial Endocarditis"[TIAB] OR "Bacterial Endocarditides"[TIAB] OR "Gram-Negative Bacterial Infections"[TIAB] OR "Gram Negative Bacterial Infection*"[TIAB] OR "Gram Positive Bacterial Infections"[TIAB] OR "Gram-Positive Bacterial Infection*"[TIAB] OR "Bacterial Skin Disease*"[TIAB] OR "Mastitis"[TIAB] OR "Bacterial Endocarditis"[OT] OR "Bacterial Endocarditides"[OT] OR "Gram-Negative Bacterial Infections"[OT] OR "Gram Negative Bacterial Infection*"[OT] OR "Gram Positive Bacterial Infections"[OT] OR "Gram-Positive Bacterial Infection*"[OT] OR "Bacterial Skin Disease*"[OT] OR "Mastitis"[OT] OR "subacute endocarditis"[TIAB] OR "Gram negative bacterium*"[TIAB] OR "Gram negative infection*"[TIAB] OR "Gram positive bacterium*"[TIAB] OR "Gram positive infection*"[TIAB] OR "bacterial skin disease*"[TIAB] OR "breast abscess*"[TIAB] OR "granulomatous mastitis"[TIAB] OR "subacute endocarditis"[OT] OR "Gram negative bacterium*"[OT] OR "Gram negative infection*"[OT] OR "Gram positive bacterium*"[OT] OR "Gram positive infection*"[OT] OR "bacterial skin disease*"[OT] OR "breast abscess*"[OT] OR "granulomatous mastitis"[OT]
#5	#3 AND #4
TOTAL	175

2.2 Scopus Search Strategy

Search strategy	Formulas
#1	“Body Piercing*” OR “Nipple Piercing*”
#2	“Ear Piercing*” OR “Lip Piercing*” OR “Navel Piercing*” OR “Tongue Piercing*” OR “Eyebrow Piercing*” OR “genital piercing*” OR “oral piercing*” OR “nose piercing*”
#3	#1 AND NOT #2
#4	“Bacterial infection*” OR “Infectio*” OR “Infection* Bacterial” OR “Bacterial Disease*” OR Bacteremia* OR Bacteri* OR “Bacterial Endocarditis” OR “Bacterial Endocarditides” OR “Gram-Negative Bacterial Infection*” OR “Gram Negative Bacterial Infection*” OR “Gram Positive Bacterial Infection*” OR “Gram-Positive Bacterial Infection*” OR “Bacterial Skin Disease*” OR Mastitis OR “subacute endocarditis” OR “Gram negative bacterium*” OR “Gram negative infection*” OR “Gram positive bacterium*” OR “Gram positive infection*” OR “breast abscess*” OR “granulomatous mastitis”
#5	#3 AND #4
TOTAL	444

2.3 Embase Search Strategy

Search strategy	Formulas
#1	('body piercing'/exp OR 'body piercing' OR 'nipple piercing'/exp OR 'nipple piercing')
#2	('ear piercing'/exp OR 'ear lobe piercing' OR 'ear piercing' OR 'tongue piercing'/exp OR 'lingual piercing' OR 'tongue piercing' OR 'lip piercing'/exp OR 'labial piercing' OR 'lip piercing' OR 'oral piercing'/exp OR 'intra-oral piercing' OR 'intraoral piercing' OR 'oral piercing' OR 'genital piercing'/exp OR 'genital piercing' OR 'nose piercing'/exp OR 'nasal piercing' OR 'nose piercing')
#3	#1 NOT #2
#4	('bacterial infection'/exp OR 'bacteria infection' OR 'bacterial infection' OR 'bacterial infections' OR 'bacterium infection' OR 'infection, bacterial' OR 'bacteremia'/exp OR 'bacillaemia' OR 'bacillemia' OR 'bacteraemia' OR 'bacteremia' OR 'bacteriemia' OR 'haemorrhagic bacteremia' OR 'hemorrhagic bacteremia' OR 'bacterial endocarditis'/exp OR 'acute bacterial endocarditis' OR 'bacterial endocarditis' OR 'bacterium endocarditis' OR 'endocarditis lenta' OR 'endocarditis, bacterial' OR 'endocarditis, infective' OR 'infectious endocarditis' OR 'infective endocarditis' OR 'subacute endocarditis'/exp OR 'endocarditis, subacute bacterial' OR 'subacute bacterial endocarditis' OR 'subacute endocarditis' OR 'gram negative bacterium'/exp OR 'gram negative bacterium' OR 'coccus, gram negative' OR 'gram negative bacillus' OR 'gram negative bacteria' OR 'gram negative coccus' OR 'gram negative germ' OR 'gram negative microbium' OR 'gram negative microorgananism' OR 'gram negative microorganism' OR 'gram negative organism' OR 'gram negative rod' OR 'gram-negative bacteria' OR 'gram positive bacterium'/exp OR 'gram positive bacillus' OR 'gram positive bacterium' OR 'bacillus, gram positive' OR 'gram positive bacteria' OR 'gram positive coccus' OR 'gram positive microbacteria' OR 'gram positive microbium' OR 'gram positive microorganism' OR 'gram positive organism' OR 'gram-positive bacteria' OR 'gram-positive endospore-forming bacteria' OR 'grampositive bacillus' OR 'microorganism, gram positive' OR 'gram negative infection'/exp OR 'gram negative infection' OR 'gram negative bacillary infection' OR 'gram negative bacterial infection' OR 'gram-negative bacterial infections' OR 'gram positive infection'/exp OR 'gram positive bacterial infection' OR 'gram positive bacterial infections' OR 'gram positive infection' OR 'gram positive infections' OR 'gram-positive bacterial infections' OR 'bacterial skin disease'/exp OR 'bacterial skin disease' OR 'bacterial skin diseases' OR 'bacterial skin infection' OR 'skin disease, bacterial' OR 'skin diseases, bacterial' OR 'mastitis'/exp OR 'breast infection' OR 'breast inflammation' OR 'chronic mastitis' OR 'comedomastitis' OR 'infection, breast' OR 'inflammation, breast' OR 'mammary infection' OR 'mastitis' OR 'mastitis chronic' OR 'breast abscess'/exp OR 'abscess, subareolar' OR 'breast abscess' OR 'subareolar abscess' OR 'granulomatous mastitis'/exp OR 'granulomatous mastitis' OR 'infection'/exp OR 'accidental infection' OR 'acute infection' OR 'autoinfection' OR 'bacterial infections and mycoses' OR 'bacteroid infection' OR 'chain of infection' OR 'focal infection' OR 'infection' OR 'infection mechanism' OR 'infection route' OR 'infection, focal' OR 'infections' OR 'infectious disease' OR 'infectivity' OR 'route of infection')
#5	#3 AND #4
TOTAL	486

2.4 Ovid/MEDLINE Search Strategy

Search strategy	Formulas
#1	exp Body Piercing/ or Body Piercing.mp. or (body piercing or body piercings or nipple piercing or nipple piercings or piercing, body or piercing, nipple or piercings, body or piercings, nipple).mp.
#2	(ear piercing or ear piercings or eyebrow piercing or eyebrow piercings or lip piercing or lip piercings or navel piercing or navel piercings or piercing, ear or piercing, eyebrow or piercing, lip or piercing, navel or piercing, tongue or piercings, ear or piercings, eyebrow or piercings, lip or piercings, navel or piercings, tongue or tongue piercing or tongue piercings).mp.
#3	#1 NOT #2
#4	((exp Bacterial Infections/ OR Bacterial Infections.mp) OR (exp Bacteremia/ OR Bacteremia.mp) OR (exp Endocarditis, Bacterial/ OR Endocarditis, Bacterial.mp) OR (exp Endocarditis, Subacute Bacterial/ OR Endocarditis, Subacute Bacterial.mp) OR (exp Gram-Negative Bacterial Infections/ OR Gram-Negative Bacterial Infections.mp) OR (exp Gram-Positive Bacterial Infections/ OR Gram-Positive Bacterial Infections.mp) OR (exp Skin Diseases, Bacterial/ OR Skin Diseases, Bacterial.mp)) OR (((((((bacterial infection.mp OR bacterial infections.mp OR infection, bacterial.mp OR infections, bacterial.mp) OR (bacteremia.mp OR bacteremias.mp)) OR (exp Infection/ OR Infection.mp) OR (bacterial endocarditides.mp OR bacterial endocarditis.mp OR endocarditides, bacterial.mp OR endocarditis, bacterial.mp)) OR (bacterial endocarditides, subacute.mp OR bacterial endocarditis, subacute.mp OR endocarditides, subacute bacterial.mp OR endocarditis lenta.mp OR endocarditis, subacute bacterial.mp OR subacute bacterial endocarditides.mp OR subacute bacterial endocarditis.mp)) OR (bacterial infection, gram-negative.mp OR bacterial infections, gram-negative.mp OR gram negative bacterial infections.mp OR gram-negative bacterial infection.mp OR gram-negative bacterial infections.mp OR infection, gram-negative bacterial.mp OR infections, gram-negative bacterial.mp)) OR (bacterial infection, gram-positive.mp OR bacterial infections, gram positive.mp OR bacterial infections, gram-positive.mp OR gram positive bacterial infections.mp OR gram-positive bacterial infection.mp OR gram-positive bacterial infections.mp OR infection, gram-positive bacterial.mp OR infections, gram positive bacterial.mp OR infections, gram-positive bacterial.mp)) OR (bacterial skin disease.mp OR bacterial skin diseases.mp OR disease, bacterial skin.mp OR skin disease, bacterial.mp OR skin diseases, bacterial.mp)) OR exp Mastitis/ or Mastitis.mp. or (exp Granulomatous Mastitis/ or Granulomatous Mastitis.mp.) or (granulomatous mastitis or mastitis, granulomatous).mp.
#5	#3 AND #4
TOTAL	288

3 Supplementary Material 3: Critical Appraisal Checklist according to Joanna Briggs Institute

3.1 Quality Assessment of Case Reports according to Joanna Briggs Institute Critical Appraisal Checklist

Author	Item 1	Item 2	Item 3	Item 4	Item 5	Item 6	Item 7	Item 8	Overall appraisal
Brook I [17]	Y	NA	Y	Y	Y	Y	NA	Y	Include
De Kleer N [18]	Y	Y	Y	Y	Y	Y	NA	Y	Include
Siddique N [29]	Y	Y	Y	Y	Y	Y	Y	Y	Include
Ceniceros A [31]	Y	Y	Y	Y	Y	Y	Y	Y	Include
Cornelissen A [32]	Y	U	Y	Y	U	Y	NA	Y	Include
Drifmeyer E [26]	Y	U	Y	Y	U	U	NA	Y	Include
Abbas K [33]	Y	U	Y	Y	Y	Y	Y	Y	Include
Pearlman M [34]	Y	Y	Y	Y	Y	Y	NA	Y	Include
Abdulrahman G [35]	Y	Y	Y	Y	Y	Y	NA	Y	Include
Trupiano J [36]	Y	U	Y	Y	Y	Y	NA	Y	Include
Lewis C [37]	Y	U	Y	Y	Y	Y	NA	Y	Include
Jacobs V [19]	Y	Y	Y	Y	Y	Y	NA	Y	Include
Jacobs V [19][a]	Y	Y	Y	Y	Y	Y	NA	Y	Include
Zardawi I [20]	Y	Y	Y	Y	Y	U	NA	Y	Include
Pendle S [21]	Y	Y	Y	Y	Y	Y	NA	Y	Include
Ochsenfahrt C [22]	Y	Y	Y	Y	U	Y	NA	Y	Include
Bader M [23]	Y	Y	Y	Y	Y	Y	NA	Y	Include
Maroun E [24]	Y	Y	Y	Y	Y	U	NA	Y	Include
Bengualid V [25]	Y	Y	Y	U	Y	U	NA	Y	Include

Item 1: Were patient's demographic characteristics clearly described?; Item 2: Was the patient's history clearly described and presented as a timeline?; Item 3: Was the current clinical condition of the patient on presentation clearly described?; Item 4: Were diagnostic tests or assessment methods and the results clearly described?; Item 5: Was the intervention(s) or treatment procedure(s) clearly described?; Item 6: Was the post-intervention clinical condition clearly described?; Item 7: Were adverse events (harms) or unanticipated events identified and described?; Item 8: Does the case report provide takeaway lessons? Y: Yes; N: No; U: Unclear; NA: Not applicable; [a]second eligible case from the same article, [b]third eligible case from the same article

3.2 Quality Assessment of Case Series according to Joanna Briggs Institute Critical Appraisal Checklist

Author	Item 1	Item 2	Item 3	Item 4	Item 5	Item 6	Item 7	Item 8	Item 9	Item 10	Overall appraisal
Shoyele O [28]	Y	Y	Y	Y	Y	Y	N	Y	Y	NA	Include
Leibman A [27]	Y	Y	Y	Y	Y	N	N	N	Y	NA	Include
Leibman A [27][a]	Y	Y	Y	Y	Y	N	N	N	Y	NA	Include
Leibman A [27][b]	Y	Y	Y	Y	Y	N	N	N	Y	NA	Include
Baker G [30]	Y	U	U	U	Y	Y	Y	Y	NA	NA	Include
Baker G [30][a]	Y	U	U	U	Y	Y	Y	Y	NA	NA	Include
Baker G [30][b]	Y	U	U	U	Y	Y	Y	Y	NA	NA	Include
Baker G [30][c]	Y	U	U	U	Y	Y	Y	Y	NA	NA	Include

Item 1: Were there clear criteria for inclusion in the case series?; Item 2: Was the condition measured in a standard reliable way for all participants included in the case series?; Item 3: Were valid methods used for the identification of the condition for all participants included in the case series?; Item 4: Did the case series have consecutive inclusion of participants?; Item 5: Did the case series have complete inclusion of participants?; Item 6: Was there clear reporting of demographics of the participants in the study?; Item 7: Was there clear reporting of clinical information of the participants?; Item 8: Were the outcomes of follow up results of cases clearly reported?; Item 9: Was there clear reporting of the presenting site(s)/clinic(s) demographic information?; Item 10: Was statistical analysis appropriate?. Y: Yes; N: No; U: Unclear; NA: Not applicable; [a]second eligible case from the same article, [b]third eligible case from the same article, [c]fourth eligible case from the same article
